# Author Correction: Phosphorylation-dependent tuning of mRNA deadenylation rates

**DOI:** 10.1038/s41594-026-01774-y

**Published:** 2026-02-18

**Authors:** James A. W. Stowell, Conny W. H. Yu, Zhuo A. Chen, Lily K. DeBell, Giselle C. Lee, Tomos Morgan, Ludwig Sinn, Sylvie Agnello, Francis J. O’Reilly, Juri Rappsilber, Stefan M. V. Freund, Lori A. Passmore

**Affiliations:** 1 MRC Laboratory of Molecular Biology, Cambridge, UK; 2 Chair of Bioanalytics, Technische Universität Berlin, Berlin, Germany; 3 Wellcome Centre for Cell Biology, University of Edinburgh, Edinburgh, UK

**Keywords:** RNA decay, Phosphorylation, Solution-state NMR, RNA-binding proteins, Intrinsically disordered proteins

Correction to: *Nature Structural & Molecular Biology* 10.1038/s41594-025-01688-1, published online 3 November 2025.

In the version of this article initially published, the name of author Giselle C. Lee was presented without the middle initial. The name has been corrected in the HTML and PDF versions of the article.

