# Supplementary material for: Phosphorylation-dependent tuning of mRNA deadenylation rates
Source: Nat Struct Mol Biol. 2025 Nov 3;33(1):63–70. doi: 10.1038/s41594-025-01688-1 (PMC12819147; doi:10.1038/s41594-025-01688-1)
Supplement: Supplementary file 1 — Supplementary Figs. 1 and 2. [file 41594_2025_1688_MOESM1_ESM.pdf]

nature structural & molecular biology

Supplementary information https://doi.org/10.1038/s41594-025-01688-1

Phosphorylation-dependent tuning of mRNA deadenylation rates

In the format provided by the authors and unedited

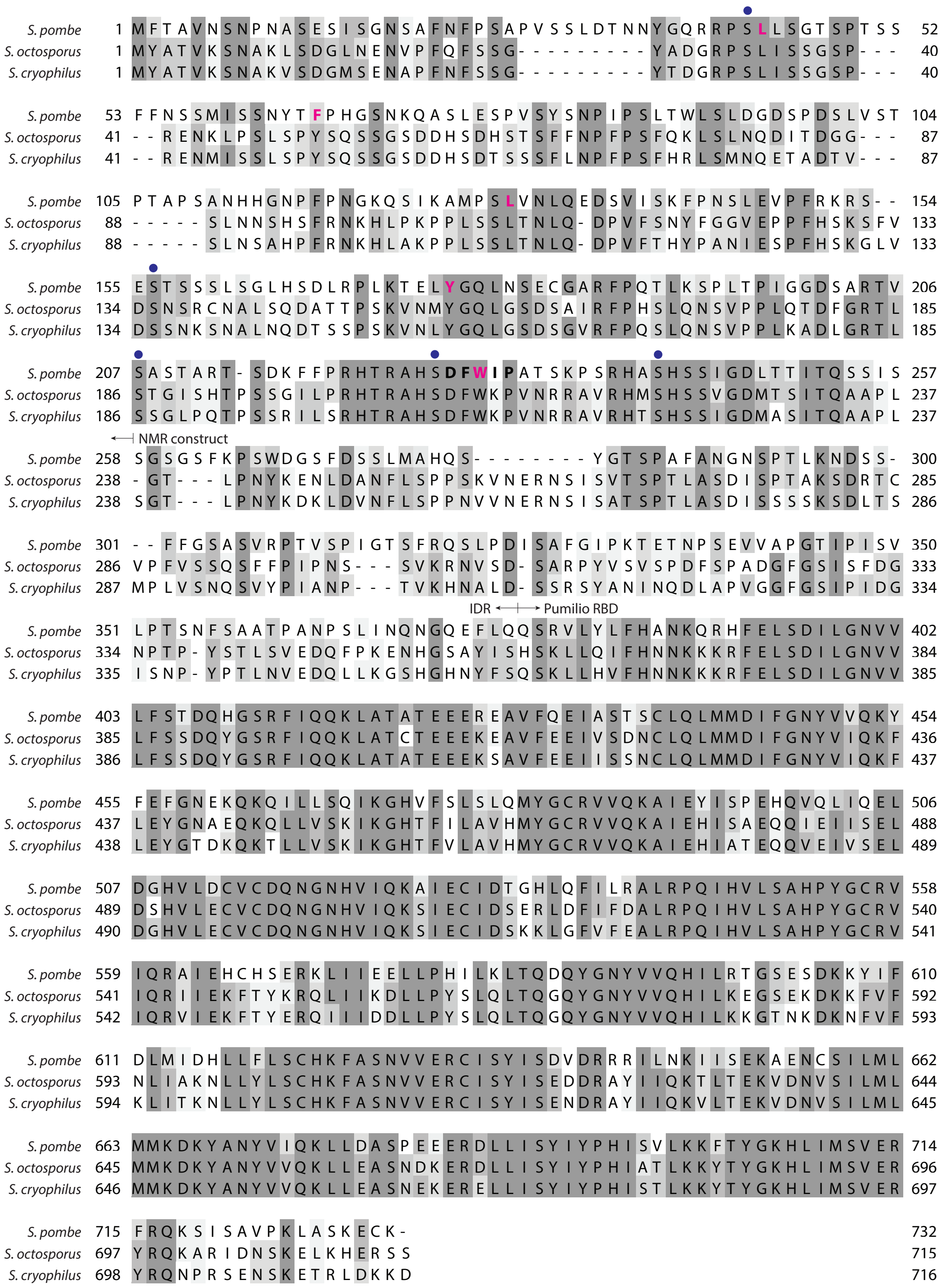

Supplementary Figure 1: Sequence alignments of Puf3. Puf3 protein homologues within the *Schizosaccharomyces* genus. IDR and Pumilio RNA binding domain (RBD) boundaries are indicated. Residues in the DFW motif are in bold (black) and residues mutated in Extended Data Fig. 3 are in bold magenta. Phosphorylation sites characterized in this study are indicated with blue dots.

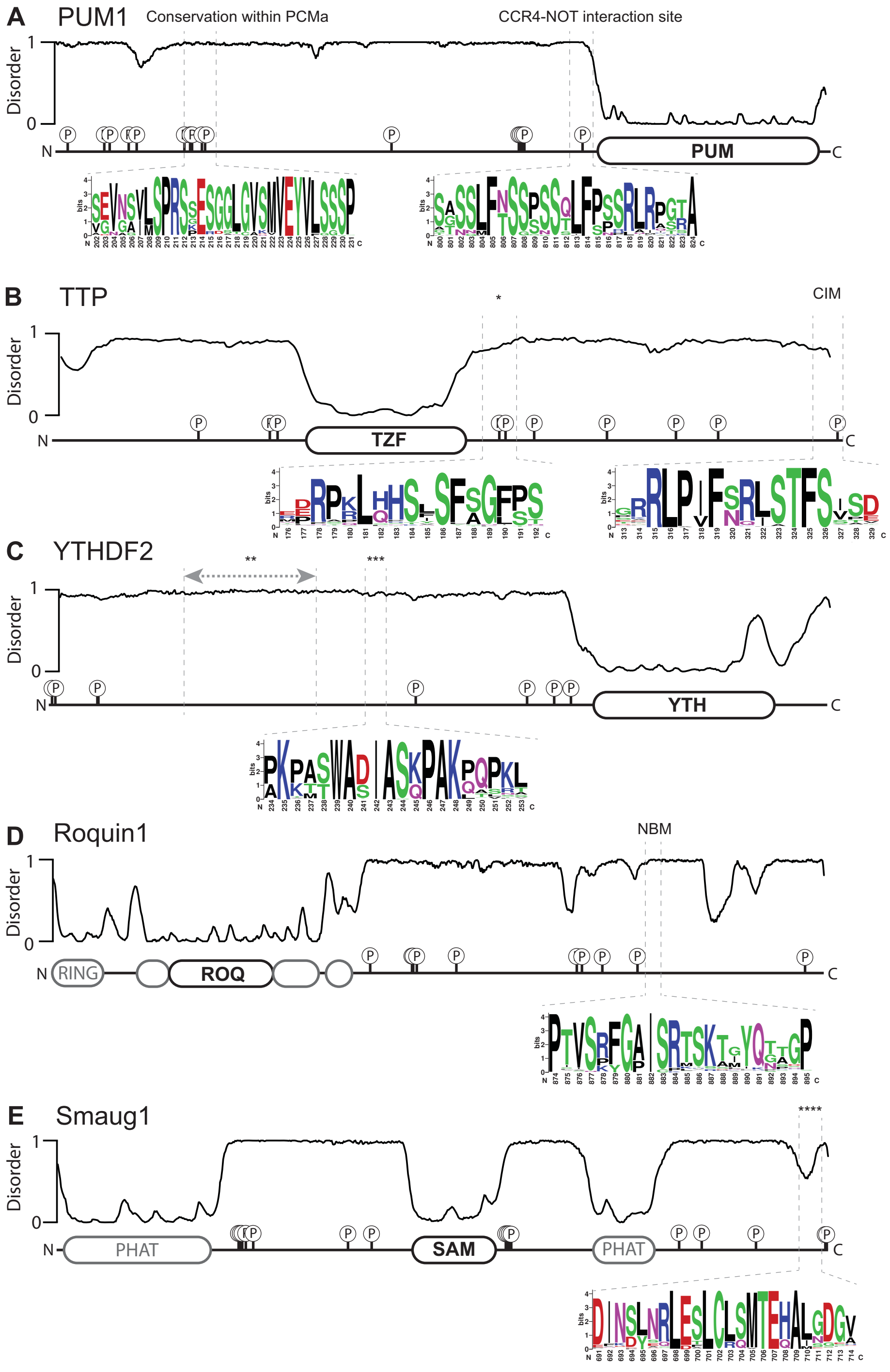

Supplementary Figure 2: Schematics of human CCR4-NOT interacting RNA-binding proteins. Disorder prediction and domain diagrams of known *H. sapiens* RNA-binding proteins that recruit the CCR4-NOT complex. Structured domains are depicted as rounded rectangles, with known RNA-binding domains in bold, with IDRs as lines. IDR boundaries are defined based on Metapredict [1]. Phosphorylation sites are annotated using data curated in PhosphoSitePlus [2]. Sequence logos show patches of conservation within IDRs which could be important for protein interactions and/or post-translational modifications, often containing conserved serines, aromatic and hydrophobic residues. Sequence logos were generated using Clustal omega alignments of 500-1000 homologous sequences from Chordates, Molluscs and Arthropods. (**A**) Pumilio homolog 1 (PUM1) contains a ~1000 amino acid IDR N-terminal to

the Pumilio RNA-binding domain. Several regions within the IDR have been implicated in interaction with CCR4-NOT. Two conserved regions of interest have been highlighted (sequence logos). The autonomously repressive Pumilio conserved motif a (PCMa) region contains several conserved phosphosites [3]. A separate region proximal to the RBD has been shown to interact with several subunits of the CCR4-NOT complex [4]. (**B**) Tristetraprolin (TTP) contains both N- and C-terminal IDRs relative to the RBD. Both have been shown to interact with CCR4-NOT. The major CCR4-NOT-interacting motif (CIM) lies close to the C-terminus of the protein [5]. Other regions within the C-terminal IDR contain both conserved aromatic and serine residues (which can be phosphorylated), indicated by an asterisk (*). (**C**) YTH domain-containing family protein 2 (YTHDF2) can directly bind the CCR4-NOT complex. A region of the N-terminal IDR spanning residues 100-200 (denoted by **) is sufficient for the interaction with the C-terminus of the NOT1 scaffold [6]. This region lies outside of the most conserved region in the IDR that contains a W-containing SLiM (shown in inset ***). This could be important for interaction with CCR4-NOT but equally could be involved in binding distinct protein partners or subcellular localization, e.g. via membrane binding. (**D**) Roquin contains multiple CCR4-NOT interacting motifs within its C-terminal IDR. Roquin has been shown to interact with both the NOT-module and CNOT9. Sequence logo shows conservation within the NOT-binding motif (NBM). (**E**) Smaug contains multiple IDRs with putative phosphoregulatory sites. Drosophila Smaug binds CCR4-NOT through an unknown interaction with the NOT module [7]. One possible candidate is a conserved C-terminal SLiM (****). This SLiM is within 10 amino acids of several C-terminal phosphosites (outside of sequence logo shown).

References

1. Emenecker, R. J., Griffith, D. & Holehouse, A. S. Metapredict: a fast, accurate, and easy-to-use predictor of consensus disorder and structure. *Biophys. J.* **120**, 4312–4319 (2021).
2. Hornbeck, P. V. et al. PhosphoSitePlus, 2014: mutations, PTMs and recalibrations. *Nucleic Acids Res.* **43**, D512–D520 (2015).
3. Weidmann, C. A. & Goldstrohm, A. C. *Drosophila* Pumilio protein contains multiple autonomous repression domains that regulate mRNAs independently of Nanos and brain tumor. *Mol. Cell. Biol.* **32**, 527–540 (2012).
4. Haugen, R. J., Arvola, R. M., Connacher, R. P., Roden, R. T.& Goldstrohm, A. C. A conserved domain of *Drosophila* RNA-binding protein Pumilio interacts with multiple CCR4–NOT deadenylase complex subunits to repress target mRNAs. *J. Biol. Chem.* **298**, 102270 (2022).
5. Fabian, M. R. et al. Structural basis for the recruitment of the human CCR4–NOT deadenylase complex by Tristetraprolin. *Nat. Struct. Mol. Biol.* **20**, 735–739 (2013).
6. Du, H. et al. YTHDF2 destabilizes m6A-containing RNA through direct recruitment of the CCR4–NOT deadenylase complex. *Nat. Commun.* **7**, 12626 (2016).
7. Pekovic, F. et al. RNA binding proteins Smaug and Cup induce CCR4–NOT-dependent deadenylation of the nanos mRNA in a reconstituted system. *Nucleic Acids Res.* **51**, 3950–3970 (2023).
