# Supplementary material for: Phosphorylation-dependent tuning of mRNA deadenylation rates
Source: Nat Struct Mol Biol. 2025 Nov 3;33(1):63–70. doi: 10.1038/s41594-025-01688-1 (PMC12819147; doi:10.1038/s41594-025-01688-1)

Fig 1b

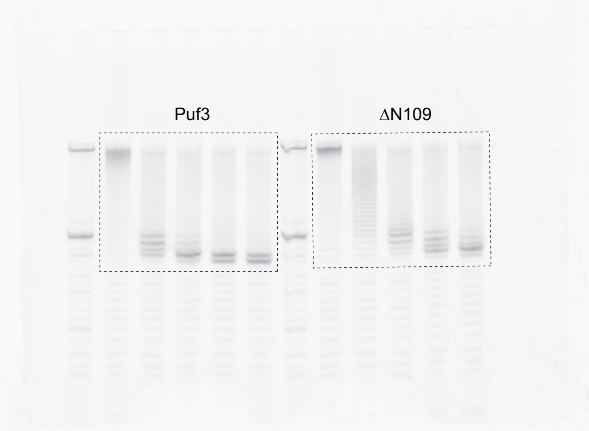

Fig 1b

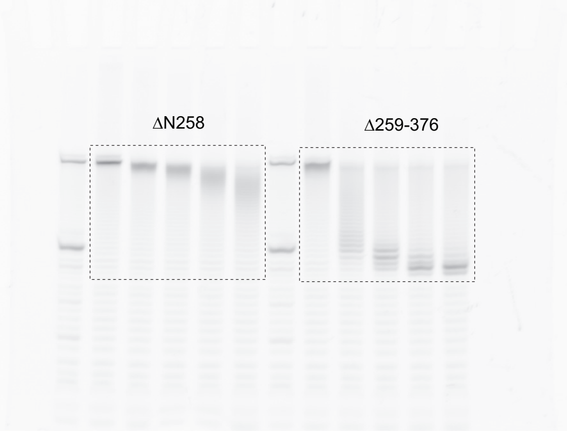

ED Fig. 1b

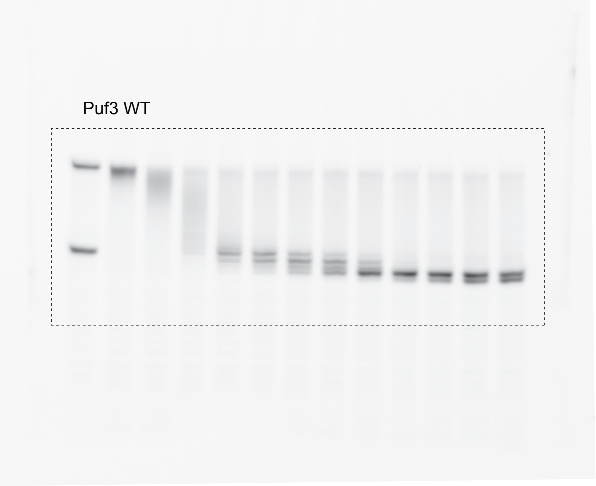

ED Fig. 1c

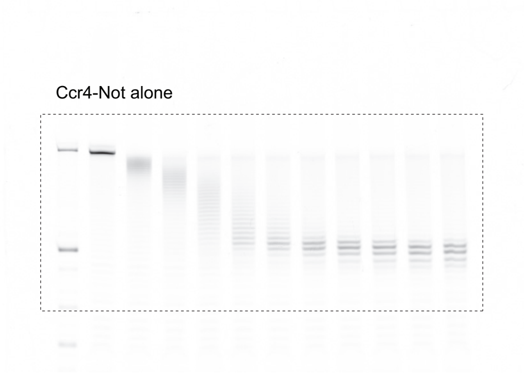

ED Fig. 1c

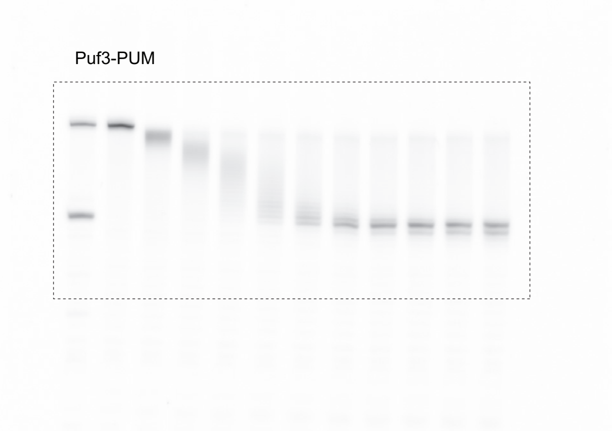

ED Fig. 1c

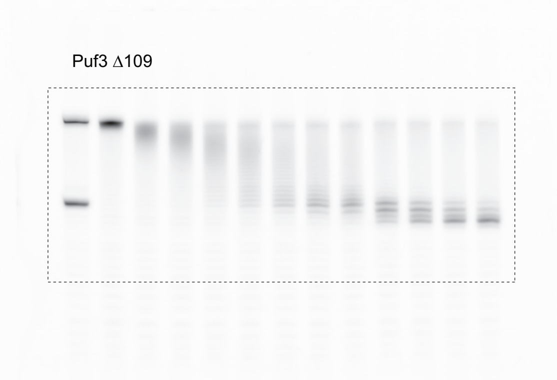

ED Fig. 1c

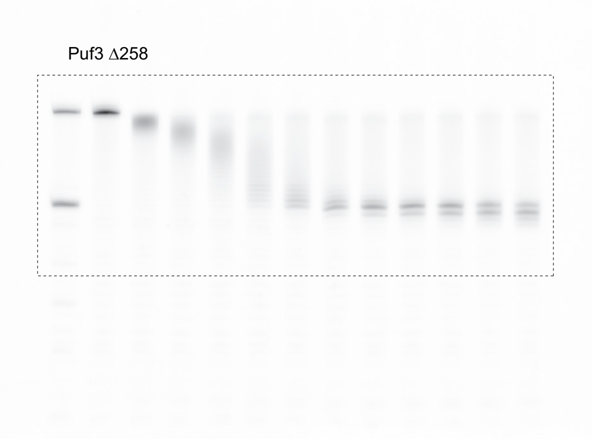

ED Fig. 1c

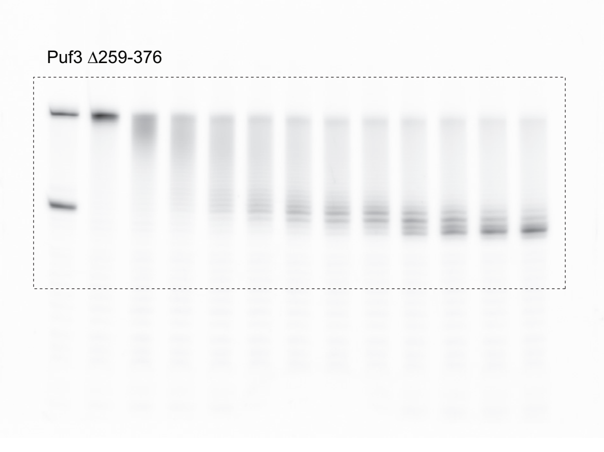

ED Fig. 3a

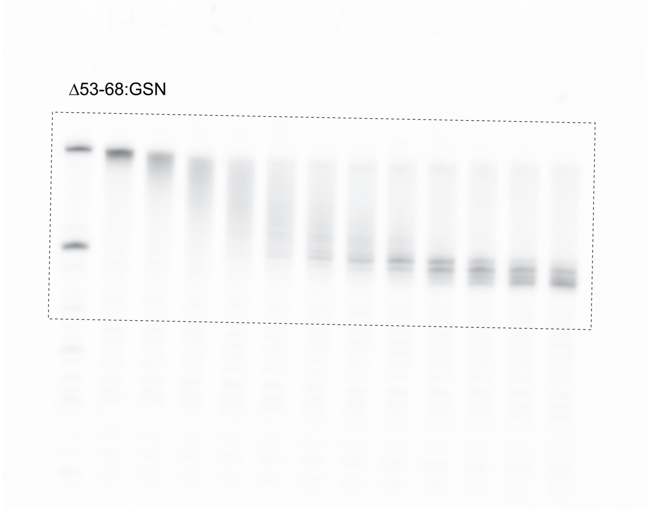

ED Fig. 3a

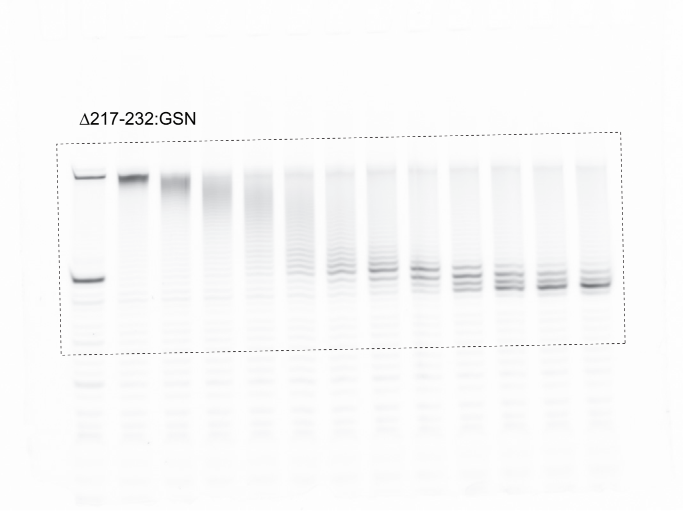

ED Fig. 3a

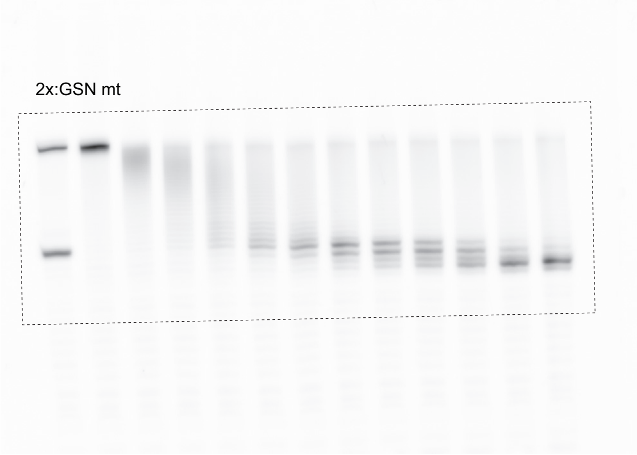

ED Fig. 3b

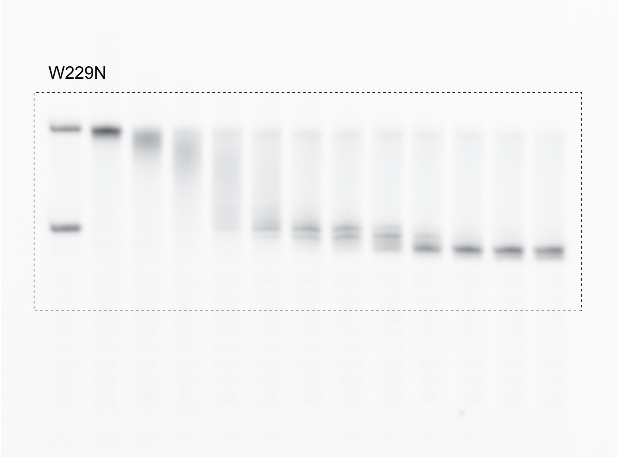

ED Fig. 3b

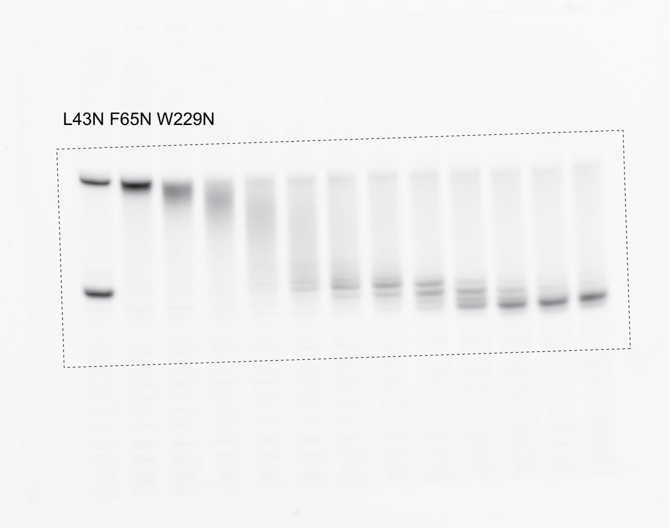

ED Fig. 3b

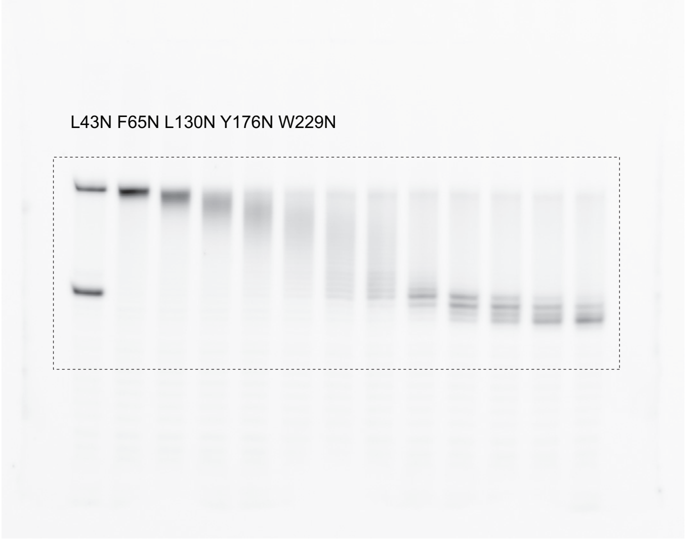

ED Fig. 4b

MBP 158-258

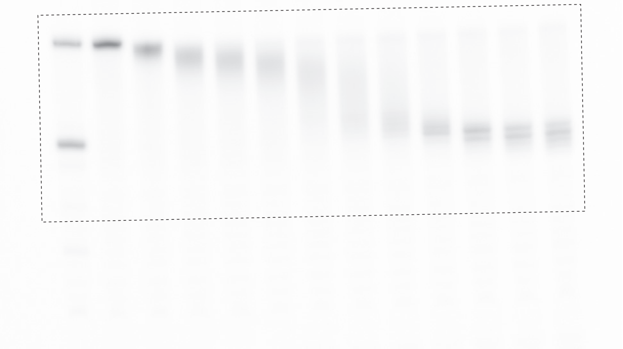

ED Fig. 4b

158-258

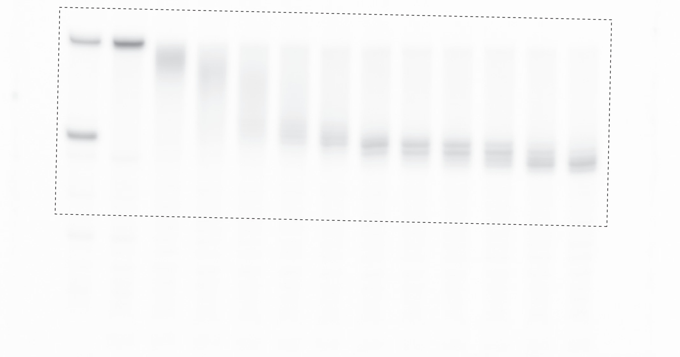

ED Fig. 4b

MBP 109-258

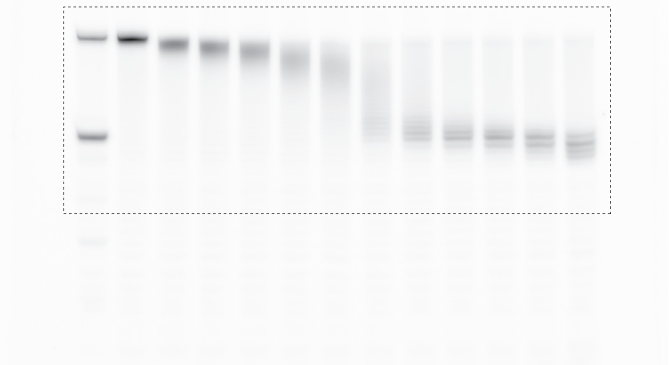

ED Fig. 4b

109-258

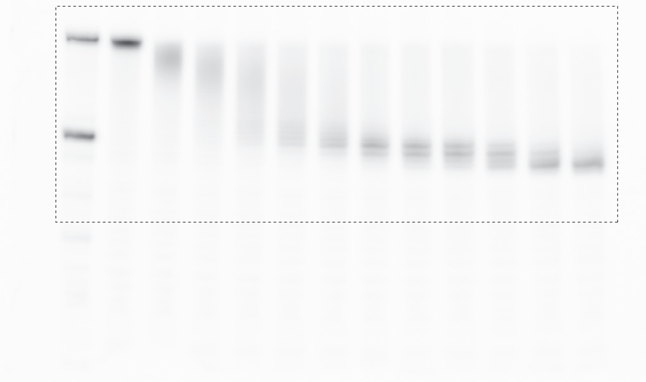

ED Fig. 4d

Input

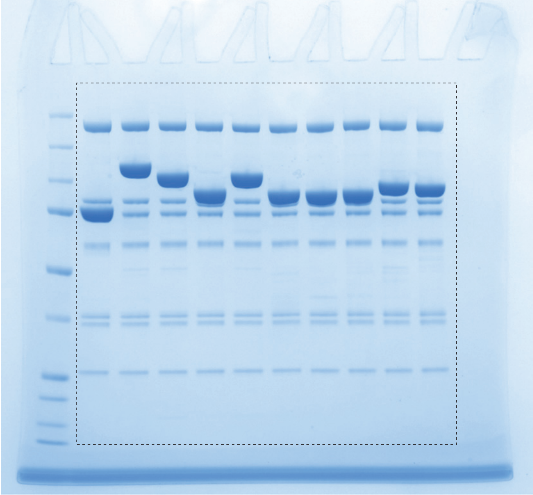

ED Fig. 4d

Pull-down

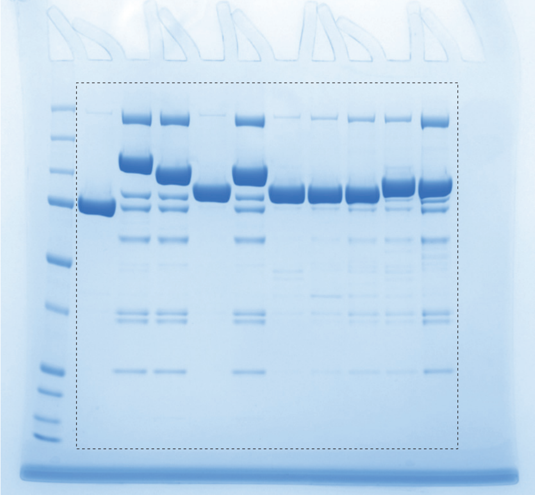

ED Fig. 6c

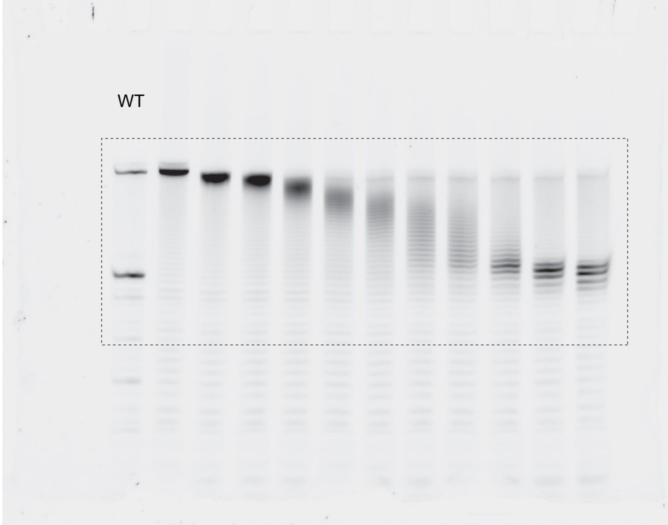

ED Fig. 6c

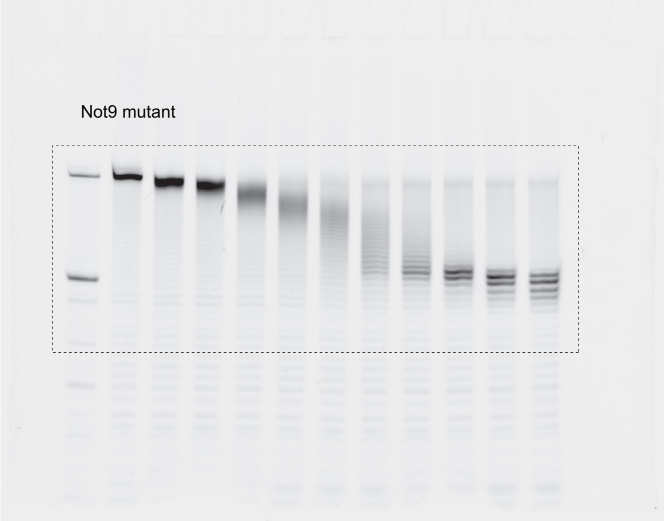

ED Fig. 6c

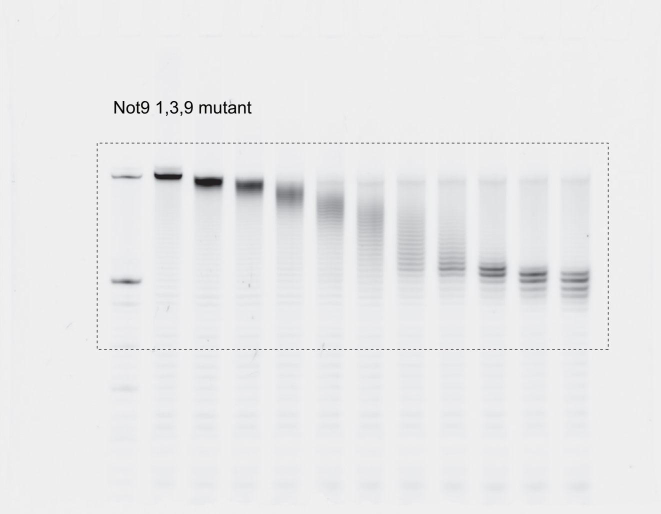

ED Fig. 6d

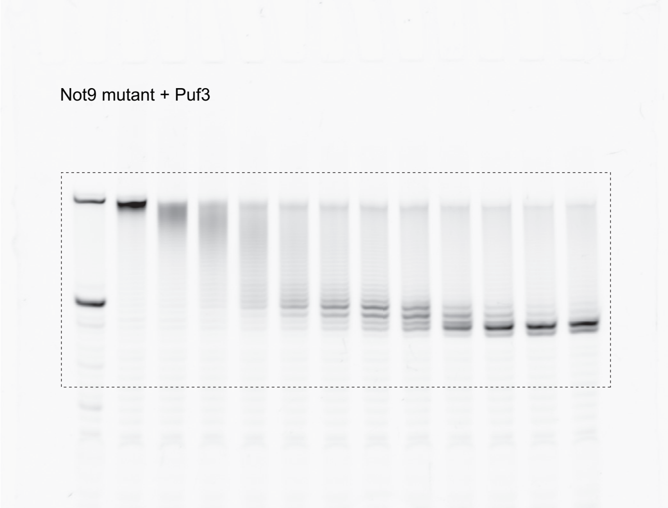

ED Fig. 6d

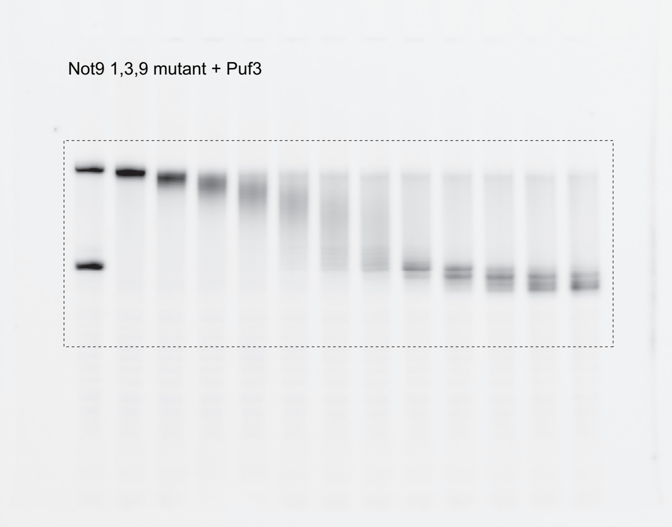

ED Fig. 8b

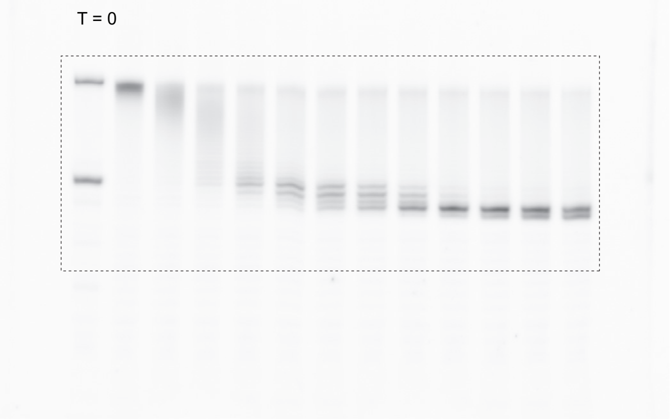

ED Fig. 8b

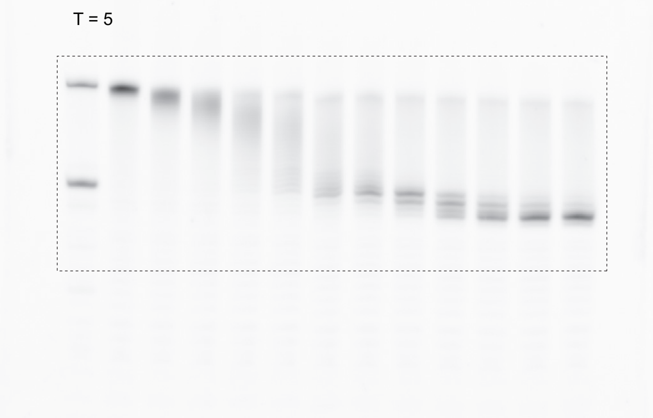

ED Fig. 8b

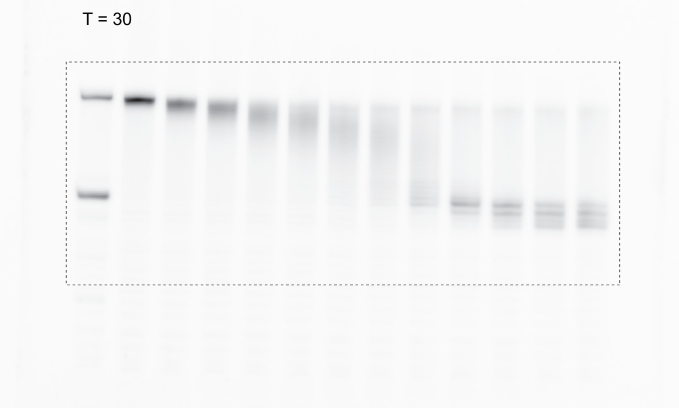

ED Fig. 8b

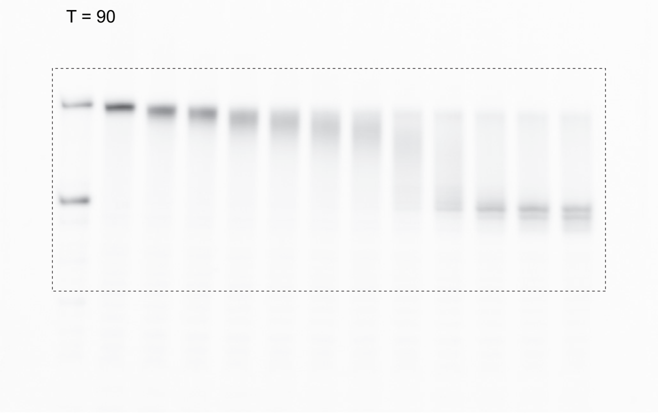

ED Fig. 9b

PUM1 (full-length)

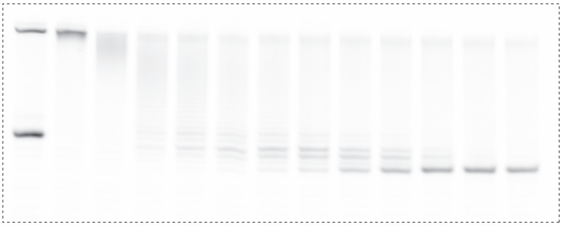

ED Fig. 9b

PUM domain (828-1186)

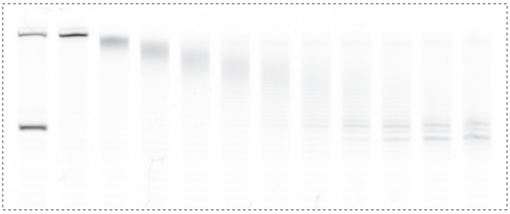

ED Fig. 9b

ΔN149

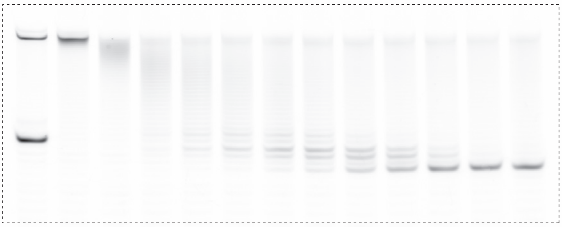

ED Fig. 9b

ΔN307

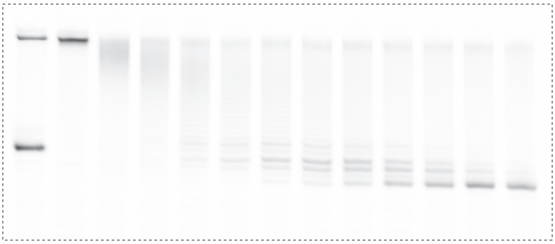

ED Fig. 9b

ΔN459

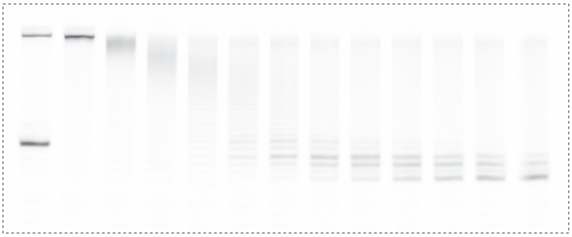

ED Fig. 9b

ΔN587

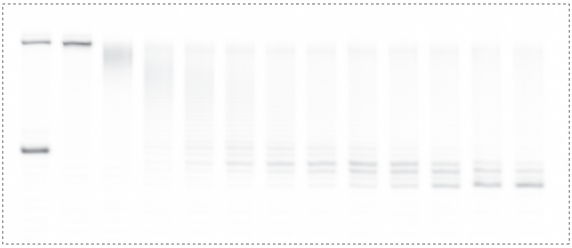

ED Fig. 10a

TTP (full-length)

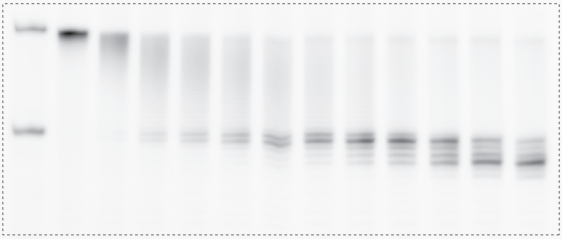

ED Fig. 10a

TTP ΔN

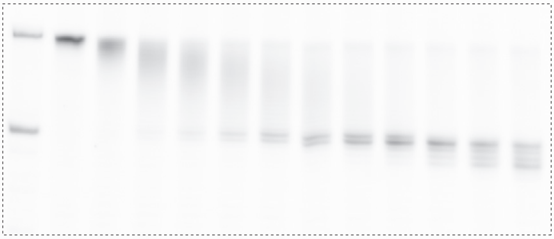

ED Fig. 10a

TTP TZF

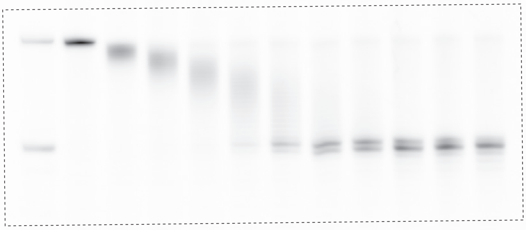

ED Fig. 10a

TTP ΔC

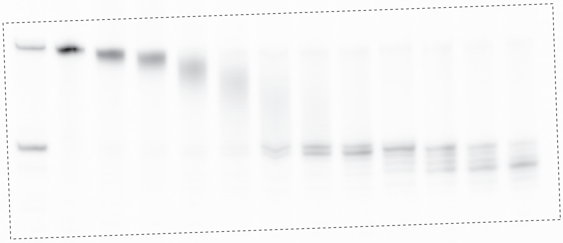

ED Fig. 10a

TTP ΔCIM

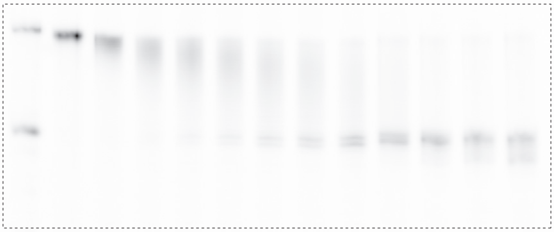

ED Fig. 10d

TTP^{3D}

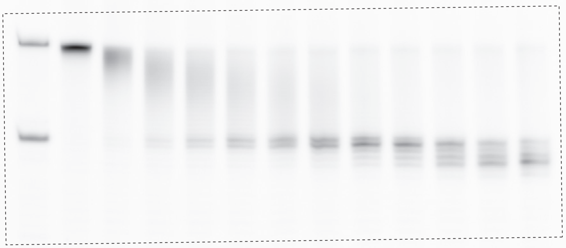

ED Fig. 10d

TTP^{6D}

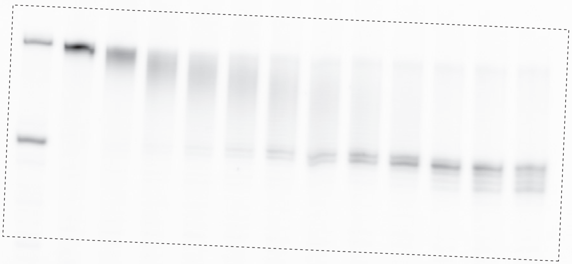

ED Fig. 10d

TTP^{9D}

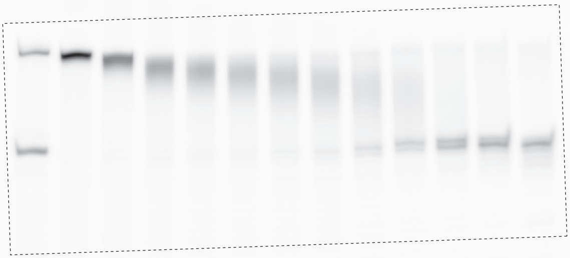

ED Fig. 10d

TTP^{12D}

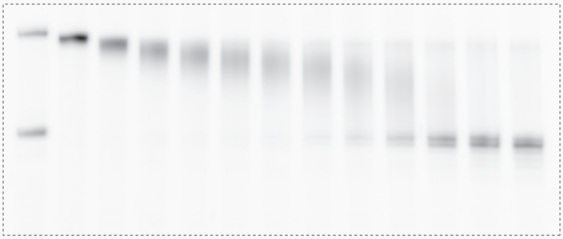

Supplement: Supplementary file 4 — Unprocessed gels and numerical data for plots. [file 41594_2025_1688_MOESM4_ESM.pdf]
